# The Learning Preferences among Nursing Students in the King Saud University in Saudi Arabia: A Cross-Sectional Survey

**DOI:** 10.1155/2017/3090387

**Published:** 2017-05-23

**Authors:** Homood A. Alharbi, Adel F. Almutairi, Eyad M. Alhelih, Abdualrahman S. Alshehry

**Affiliations:** ^1^College of Nursing, King Saud University, Riyadh, Saudi Arabia; ^2^King Abdullah International Medical Research Center, King Saud bin Abdulaziz University for Health Sciences, Riyadh, Saudi Arabia

## Abstract

**Objective:**

The present study aimed to identify the most common learning preferences among the nursing students in Saudi Arabia and to investigate the associations of certain demographic variables with the learning preferences.

**Methods:**

All the undergraduate nursing students in the nursing college were requested to participate in this descriptive cross-sectional study. An Arabic version of the Felder-Silverman learning style model (FSLSM) questionnaire was used to examine the learning preferences among undergraduate nursing students.

**Results:**

A total of 56 (43%) completed questionnaires were included in the final analysis. Results of the present study indicate that the most common learning preferences among the nursing students were visual (67.9%), followed by active (50%) and sequential (37.5%) learning preferences. The verbal style was the least common learning preference (3.6%) among the nursing students. There was no association between gender and learning preferences (*p* > .05).

**Conclusion:**

The present study concluded that the visual, active, and sequential styles are the commonest learning preferences among the nursing students. The nursing educators should emphasize the use of this information in their teaching methods to improve learning skills among the nursing students.

## 1. Introduction

The concept of learning styles has developed among professional educators at all stages of the educational organization [1]. Some students seem to learn better when information is presented through words known as verbal learners, whereas others seem to learn better when it is presented through pictures known as visual learners [2]. Some learners are active and prefer to work in a group, while others are reflective who prefer independent learning [1, 3]. There is evidence that different learning styles affect academic achievements. For instance, Surjono examined the effects of different learning styles on undergraduate student achievement and the findings suggested that students learn better when there is a match between students' learning styles and their instructor's teaching styles [4]. Similarly, Ahmad et al. [5] reported a significant relationship between students learning preference and the academic achievement. Nevertheless, Çakiroglu reported many variables that can affect academic achievement which include learning styles and studying habits [6].

In a previous study [7], in problem-based learning (PBL) class sessions, students who preferred active learning participated in the group and engaged in solving the problems, while reflective learners tend to work independently and used previously acquired information more often compared to active learners [7]. However, there were no statistical differences between the two styles (active style versus reflection style) in regard to the exam scores and genders [7]. Similarly, Nuzhat et al. [8] reported different learning preferences among undergraduate medical students in Saudi Arabia.

Research in the field of teaching and learning has suggested that students have a variety of learning methods. For example, some students remember best if they hear materials, and therefore they prefer verbal form of learning while others remember best if they see materials, and therefore they prefer visual form of learning. In the learning process, student uses different ways and resources to improve in their learning, which is known as learning styles [9, 10]. The learning style is defined as “the biologically and developmentally imposed set of characteristics that make the same teaching method wonderful for some and terrible for others” [11]. In addition, Keefe defines learning style as the “composite of characteristic cognitive, affective, and physiological characters that serve as relatively stable indicators of how a learner perceives, interacts with, and responds to the learning environment” [12]. Learning preferences are defined as individual's specific patterns of strength, weakness, and preferences in processing, absorbing, and retrieving information [13]. Learning pattern is defined as selective, repetitive character presented in the student's learning responses according to the learning demands [14]. In other words, mode of obtaining knowledge is known as learning style whereas preferred mode of obtaining knowledge is called learning style preference [15].

Learning styles are vital aspects in education psychology in any discipline, characterized by perpetual affective and cognitive behaviors which indicate how each individual communicates in learning environments or situations [16, 17]. Previous studies published various theoretical models indicating learning styles and their psychometric properties. Felder and Silverman's [18] and Kolb's learning [19] models are the most common published learning models in nursing literature. Most explanation of individual learning styles classify person into only a small number of groups. However, Felder and Silverman's learning model advances further: this model distinguishes learning preferences on four-dimensional levels, therefore allowing robust education system to conceive a learning model that is more custom-fit to learner preferences. Felder indicates that students with obvious learning preferences may find difficulty in coping with the information given via methods not compatible with their preferences [18]. Felder and Silverman presented four categories of learning preferences as follows: input (visual/verbal), perception (sensing/intuitive), processing (active/reflective), and understanding (sequential/global). The Index of Learning Style (ILS) designed by Felder and Silverman [18] comprises 44 items to assess the individual's learning preferences. Scores for each domain identified by adding the number of items answered in each of the two preferences. Scores ranking 1–3 show that participant has fair preference to that domain, with 5–7 moderate and 9–11 a strong learning preference to that domain. Felder-Silverman learning instruments were originally developed for the engineering students but can be utilized for any discipline including nursing. A previous study adapted the Felder-Silverman learning instruments into Turkish and examined the learning preferences among the students of different fields of science including health science [20]. Reviewing the body of literature revealed various styles and techniques in teaching and thus that there are also individual learning preferences [21]. For example, some people may have specific preferences such as visual, auditory, verbal, physical, logical, interpersonal, or intrapersonal preferences, over the other learning preferences [22]. Many people may have multiple learning preferences; however, one preference can be more dominant [23]. It is noteworthy that these learning preferences can significantly affect the students' academic achievements [2, 24].

Previous studies indicated the beneficial effect of matching teaching and learning styles on students learning. A meta-analysis conducted by Lovelace [25] reported that instruction matched with individual's learning styles improved academic achievement and enhanced attitudes towards learning. In addition, Stevenson and Dunn [26] suggested that student may learn more rapidly and effectively if preferred learning style was used. Similarly, Jessee et al. [27] reported the benefits of preferred learning and teaching styles in dental students. Therefore, it is imperative for educators to incorporate different learning styles in their teaching plan to accommodate students' preferences and ultimately result in a better outcome [6]. Many scholars argue that delivering knowledge in the students' preferred learning styles can increase the motivation to learn [8]. In line with this theory, Felder and Silverman claimed that learners who prefer a specific learning style could have difficulties if teaching styles that differ from their preferred ones [18].

Nursing education in Saudi Arabia has seen many changes since it was started. First nursing course of one-year program for males was started by the ministry of health in the year 1960 [28]. After that it was upgraded to three-year diploma in nursing program for both males and females [28, 29]. It was equivalent to a licensed nursing practice in the United States [29]. In the year 1976, first two colleges for female nursing were established at King Saud University, Riyadh, and King Abdul-Aziz University, Jeddah [28]. Both the schools started a bachelor in nursing program. Gradually, these universities also started postgraduate degrees in nursing. In addition, King Abdul-Aziz University in cooperation with a British university had started a Ph.D. program in nursing in the year 1996 in Saudi Arabia [30]. An independent college of nursing was started in King Saud University in the year 2004. The college of nursing provides quality education and runs various courses to fulfil the need of nursing professional in Saudi Arabia [31]. A competent and well-educated nursing workforce is vital to meet the demand of a growing population. Therefore, an effective education system at undergraduate and postgraduate levels is required within the universities [32–34].

Previous studies have examined the learning preferences among students worldwide and nationwide [20, 23, 35–39]. However, none of these studies have examined the learning preferences among undergraduate nursing students in the Saudi Arabia. Hence, this study aimed to determine the learning preferences among undergraduate nursing students at King Saud University, Saudi Arabia. The present study would identify the optimal ways to satisfy the different learning styles as preferred by the students. In addition, the present study would help students to know their own preferred learning styles and how to enhance their learning outcomes using their preferred learning styles.

## 2. Methods

This is a descriptive cross-sectional study that examines the learning preferences among Saudi nursing students. Descriptive cross-sectional studies are an important method to evaluate the proportion of a nursing population with learning preferences [14] in Saudi Arabia. Descriptive cross-sectional studies are useful for indicating preliminary evidence for a causal relationship. A convenience sample of undergraduate nursing students (from the 1st to 8th academic levels) from the college of nursing, King Saud University, Saudi Arabia, were requested to participate in this study. Approval of the study was obtained from the Institutional Review Board, King Saud University. In addition, each participant was requested to provide a written informed consent before starting the study.

### 2.1. Survey Instruments

An Arabic version of the Felder-Silverman learning style model (FSLSM) questionnaire was used in this survey to examine the preferred learning styles among undergraduate nursing students [40]. It is a 44-item self-report scale with a two-point response choice (first pole, +1 = answer a, and second pole, −1 = answer b). There are four dimensions in FSLSM. Each dimension indicates that each learner has a specific preference for learning. The first dimension distinguishes between an active and reflective learner, the second dimension distinguishes between sensing versus intuitive learners, whereas the third dimension differentiates between visual and verbal learners. In the fourth dimension, the learners are categorized according to their understanding. Each dimension is measured in eleven questions. Thus, each dimension score ranges from −11 to +11. A high score in each dimension represents the preference for the first pole of each dimension such as active, sensing, visual, or sequential dimension, whereas a low score represents the second pole of each dimension such as reflective, intuitive, verbal, or global dimension. The questionnaire is valid and reliable tool to measure the learning preferences [40, 41].

### 2.2. Data Collection

Two independent research assistants (RAs) were recruited by the principal investigator to collect the data. One RA collected the data from the female students, while the other RA collected the data from the male students. An Arabic version of the Felder-Silverman learning style model (FSLSM) questionnaire was used in this survey to examine the preferred learning styles among undergraduate nursing students [40]. The survey questionnaire was distributed to the participants in a sealed envelope. Participants were requested to complete the questionnaire and return back to RA in a sealed envelope. In addition, all the participants were requested to complete a demographic questionnaire including their age, gender, academic level, GPA, work status, and previously earned college degree. Participants had an option to return blank or incomplete surveys in the envelopes. Thus, the RAs would have no knowledge about who had participated in the study and who had not.

### 2.3. Statistical Analysis

Data was analyzed using the Statistical Package for the Social Sciences (SPSS, version 23). Descriptive statistics including frequency and percentage were calculated to characterize the study sample. The Pearson Chi-Square analysis was done to investigate association of participants' learning preferences with demographic variables including gender, academic achievements (GPA), previously earned degree(s), employment status, and working hours/week. A level of statistical significance was set at *p* < .05.

## 3. Results

The present study had a response rate of 66% (86 of 130 nursing students). Thirty questionnaires were excluded due to incomplete information. A total of 56 (43%) completed questionnaires were included in the final analysis.  Table 1 shows the participant's characteristics. Results of the present study indicate that the most common learning preferences among the nursing students was visual (67.9%), followed by active (50%) and sequential (37.5%) learning preferences, as shown in the Table 2. The verbal style was the least common learning preference (3.6%) among the nursing students, as shown in Table 2.

Distribution and association of participants' learning preferences according to gender, academic achievement, qualification, employment status, and working hours were presented in Table 3. There was no association between gender and learning preferences. In the present study, the academic achievements were used to divide the participants into two categories: high achievers with a GPA from 4 to 5 and low achievers with a GPA below 4. The results of present study indicated that the academic achievement of the participants was not associated with the learning preferences among the nursing students. In addition, there was no association between previously earned degree and learning preferences. However, there was a significant association between employment status and learning preferences (*p* = .022). Similarly, there was a significant association between the working hours per week and learning preferences (*p* = .003).

## 4. Discussion

Learning is the process in which learner takes an active part [20]. Hence, learner recognizes and learns his/her weaknesses and strengths. In the individual learning style, each individual gets to know how he/she realizes and perceives the learning process. To develop an attitude and the ability of lifelong learning, individuals need to know about their learning style and they should know their strengths and weaknesses [42].

Learning is the most important factor for all the fields of education. Like other fields, it is important for nursing educators to recognize the most common learning preferences in nursing students. Identification of the most common learning preferences in nursing students will help educators to learn more about their students and improves their mode of teaching and adapts a variety of learning styles. In addition, acknowledging all these information will help them to develop more effective curriculum design [43]. In a previous study, Rassool and Rawaf [44] reported a serious consequence of incongruity between learning preferences and teaching style. Students may become uninterested, might be discouraged, perform poorly in the tests, and finally may give up the course [18, 45, 46]. The use of innovation and technological clinical environment and expanded autonomy of nurses have suggested more advancement in learning strategies and teaching methods with other learning opportunities in nursing education training [44]. Divergence in the nursing profession has brought new demands to the nursing education with entrance of greater number of diverse and grown-up students. In addition, increased number of heterogeneous group of students has appeared in different skills, aptitude, and competence [44]. These difficulties should be addressed through organized way in a variety of teaching and learning activities. Furthermore, to provide a positive and effective learning in nursing education, nursing teachers have designed a variety of learning and teaching techniques based on their teaching styles [44]. Moreover, it is well known that learning styles influences student learning, and a significant association was found among learning preferences including personality, gender, clinical education, academic achievement, and student retention [47–50]. Thus, learning preference becomes one of the important factors to improve student learning and increase their skills, aptitude, and competency in nursing education. In addition, knowledge about the learning preferences of students can strengthen learning for the individuals who are poor performers in their academic studies. The individuals who are “at risk” might be supported with individual classes where custom-made additional learning program can be developed and introduced [44].

Therefore, the present study aimed to identify the most common learning preferences among the nursing students in Saudi Arabia and to investigate the associations of certain demographic variables with the learning preferences. The most common learning preference in the sample was visual (67.9%). Similarly, in previous studies, visual learning was the most preferred among the science students [51–53]. The results of present study also indicated that the common learning preferences in four dimensions are as follows: active is more preferred than reflective style, sensing is more preferred than intuitive style, visual is more dominant than verbal style, and sequential is more preferred than global style. A previous study reported similar learning preferences among the students of various fields of science in Turkey [20].

In the present study, gender was not associated with the learning preferences. The most common learning preferences among the male and female student was visual learning, followed by active and sequential learning. Similarly, previous studies reported a nonsignificant gender differences among medical, dental, and midwifery students [43, 54, 55]. In addition, a recent study reported nonsignificant gender differences among dental students in Saudi Arabia [23]. In contrast, another study reported significant gender differences among the physiology students [56]. Hallin [57] reported significant differences between men and women in their learning preferences in the nursing education. Women were highly motivated, auditory, tactile, and kinesthetic and preferred structure and mobility than men. Therefore, nursing educators are suggested to take into account possible differences in women's and men's learning approaches.

Academic achievement is one of the predictors of student's performance and learning ability. In the present study, the GPA score was used as an indicator of academic achievements of the students. In this study, there was a nonsignificant association between the GPA score and their learning preferences. Students with a higher GPA were among those who preferred mostly active learning, while students with a lower GPA were among those who preferred mostly visual learning. However, the differences in the learning preferences were statistically not significant (*p* > .05). Similarly, Baykan and Naçar [54] reported a nonsignificant association between GPA and the learning preferences. A nonsignificant relationship between learning preferences and academic achievement was maybe due to the fact that GPA was the single indicator of academic performance. In contrast, another study reported a significant association between the GPA score and the learning preferences in the dental students in Saudi Arabia [23].

In addition, the present study investigated the association of the employment status and working hours per week with the learning preferences in the nursing students. Interestingly, these variables were significantly associated with the learning preferences. However, these variables were not investigated previously.

The educational implication of the present study would be that the nursing educator should emphasize the use of visual modalities such as pictorial modalities, illustrations to facilitate learning process in nursing students. In addition, student should be encouraged to take active participation such as discussion and physical activity to acquire knowledge. Furthermore, the type of information should be presented in a sensory form such as sound and sights to improve learning skills. Moreover, nursing educator should evaluate their teaching and learning style preferences with the goal that they know their unintended bias towards specific learning and teaching strategies. This self-awareness may empower educators to expand their learning and teaching activities relying upon the learning settings, for example, clinical, classroom, or laboratory-based setting. However, further research is warranted in nursing education to identify whether matching of student learning preferences and educator learning styles is more or less effective in improving teaching learning strategies and learning skills.

The present study had several potential limitations. First of all, due to a small sample size the results of the present study cannot be generalized to all the nursing students. In addition, sample was from one university only, which further limits its generalizability. Due to cross-sectional nature of the study, a causal relationship could not be made. In the future, studies with larger sample size and variety of geographical regions are recommended to determine if the present findings are applicable to whole target population.

## 5. Conclusion

The present study concluded that the learning preferences of the nursing students were visual, active, and sequential. Gender, academic achievement, and previously earned degrees were not associated with the learning preferences. However, employment status and working hours were associated with the learning preferences in the nursing education. Educators have to be aware of their students' preferred learning styles. This could help in advancing the science of nursing education in Saudi Arabia and may aid in building and creating evidence-based culture.

## Figures and Tables

**Table 1:** Participant's characteristics.

*N* = 56	Number	Percentage
Gender		
Male	41	73.2
Female	15	26.8

Academic achievements (GPA)		
High achievers (4-5)	23	41.1
Low achievers (below 4)	33	58.9

Previously earned degree(s)		
High school	32	57.1
Diploma	24	42.9

Employment status		
Yes	26	46.4
No	30	53.6

Working hours/week		
40–45 hours	34	60.7
46–50 hours	22	39.3

**Table 2:** The distribution of learning style.

*N* = 56	Number	Percentage
Active/reflective		
Balanced	22	39.3
Active	28	50.0
Reflective	6	10.7

Sensing/intuitive		
Balanced	40	71.5
Sensing	11	19.6
Intuitive	5	8.9

Visual/verbal		
Balanced	16	28.5
Visual	38	67.9
Verbal	2	3.6

Sequential/global		
Balanced	31	55.4
Sequential	21	37.5
Global	4	7.1

**Table 3:** Distribution and association of participants' learning styles according to gender, academic achievement, qualification, employment status, and working hours.

*N* = 56	Active	Reflective	*p*	Sensing	Intuitive	*p*	Visual	Verbal	*p*	Sequential	Global	^*∗*^ *p*
	*N* (%)	*N* (%)		*N* (%)	*N* (%)		*N* (%)	*N* (%)		*N* (%)	*N* (%)	
Gender												
Male	21 (37.5)	4 (7.1)	.914	8 (14.2)	3 (5.3)	.774	28 (50)	1 (1.7)	.748	16 (28.5)	2 (3.5)	.546
Female	7 (12.5)	2 (3.5)		3 (5.3)	2 (3.5)		10 (17.8)	1 (1.7)		5 (8.9)	2 (3.5)	

Academic achievements (GPA)												
High achievers (4-5)	15 (26.7)	3 (5.3)	.079	6 (1.7)	2 (3.5)	.595	14 (25)	2 (3.5)	.202	8 (14.2)	1 (1.7)	.698
Low achievers (below 4)	13 (23.3)	3 (5.3)		5 (8.9)	3 (5.3)		24 (42.8)	0 (0)		13 (23.2)	3 (5.3)	

Previously earned degree(s)												
High school	15 (26.7)	4 (7.1)	.818	7 (12.5)	2 (3.5)	.673	25 (44.6)	1 (1.7)	.155	10 (17.8)	3 (5.3)	.469
Diploma	13 (23.2)	2 (3.5)		4 (7.1)	3 (5.3)		13 (23.2)	1 (1.7)		11 (19.6)	1 (1.7)	

Employment status												
Yes	13 (23.2)	2 (3.5)	.769	6 (1.7)	3 (5.3)	.634	15 (26.7)	1 (1.7)	.300	14 (25)	0 (0)	.022^*∗*^
No	15 (26.7)	4 (7.1)		5 (8.9)	2 (3.5)		23 (41.1)	1 (1.7)		7 (12.5)	4 (7.1)	

Working hours/week												
40–45 hours	19 (33.9)	2 (3.5)	.285	5 (8.9)	4 (7.1)	.385	25 (44.6)	1 (1.7)	.528	7 (12.5)	4 (7.1)	.003^*∗*^
46–50 hours	9 (16.1)	4 (7.1)		6 (1.7)	1 (1.7)		13 (23.2)	1 (1.7)		14 (25)	0 (0)	

^*∗*^Pearson Chi-Square test (significant if *p* < .05).
